# Local regulation of antral follicle development and ovulation in monovulatory species

**DOI:** 10.1590/1984-3143-AR2022-0099

**Published:** 2023-01-06

**Authors:** Fabiane Pereira de Moraes, Daniele Missio, Jessica Lazzari, Monique Tomazele Rovani, Rogério Ferreira, Paulo Bayard Dias Gonçalves, Bernardo Garziera Gasperin

**Affiliations:** 1 Programa de Pós-graduação em Veterinária, Faculdade de Veterinária, Universidade Federal de Pelotas, Capão do Leão, RS, Brasil; 2 Rede FiBRA-RS - Fisiopatologia e Biotécnicas da Reprodução, Santa Maria, RS, Brasil; 3 Universidade Federal de Santa Maria, Santa Maria, RS, Brasil; 4 Universidade Federal do Rio Grande do Sul, Porto Alegre, RS, Brasil; 5 Faculdade de Zootecnia, Universidade do Estado de Santa Catarina, Chapecó, SC, Brasil

**Keywords:** oocyte, deviation, transforming growth factors

## Abstract

The identification of mutations in the genes encoding bone morphogenetic protein 15 (BMP15) and growth and differentiation factor 9 (GDF9) associated with phenotypes of sterility or increased ovulation rate in sheep aroused interest in the study of the role of local factors in preantral and antral folliculogenesis in different species. An additive mutation in the BMP15 receptor, BMPR1b, which determines an increase in the ovulatory rate, has been introduced in several sheep breeds to increase the number of lambs born. Although these mutations indicate extremely relevant functions of these factors, the literature data on the regulation of the expression and function of these proteins and their receptors are very controversial, possibly due to differences in experimental models. The present review discusses the published data and preliminary results obtained by our group on the participation of local factors in the selection of the dominant follicle, ovulation, and follicular atresia in cattle, focusing on transforming growth factors beta and their receptors. The study of the expression pattern and the functionality of proteins produced by follicular cells and their receptors will allow increasing the knowledge about this local system, known to be involved in ovarian physiopathology and with the potential to promote contraception or increase the ovulation rate in mammals.

## Introduction

**Figure 1:**
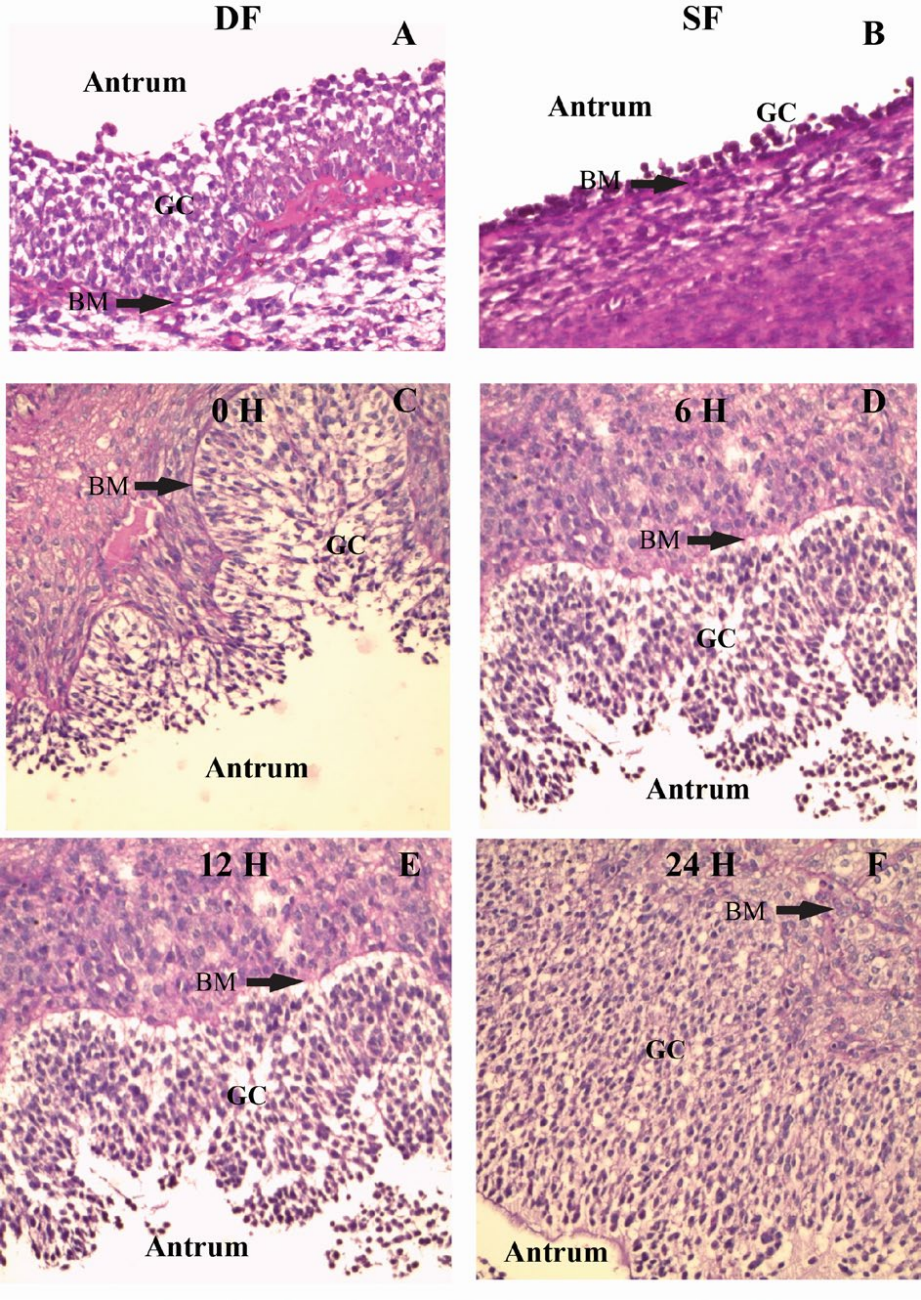
Representative images (PAS staining) of follicles used to characterize gene expression. (A) Dominant (healthy) and (B) subordinate (atretic) follicles collected after follicular deviation; (C), (D), (E), (F) preovulatory follicles collected 0, 6, 12 and 24 h after GnRH treatment, respectively. DF: dominant follicle; SF: subordinate follicle; GC: granulosa cells; BM: basal membrane.

**Figure 2:**
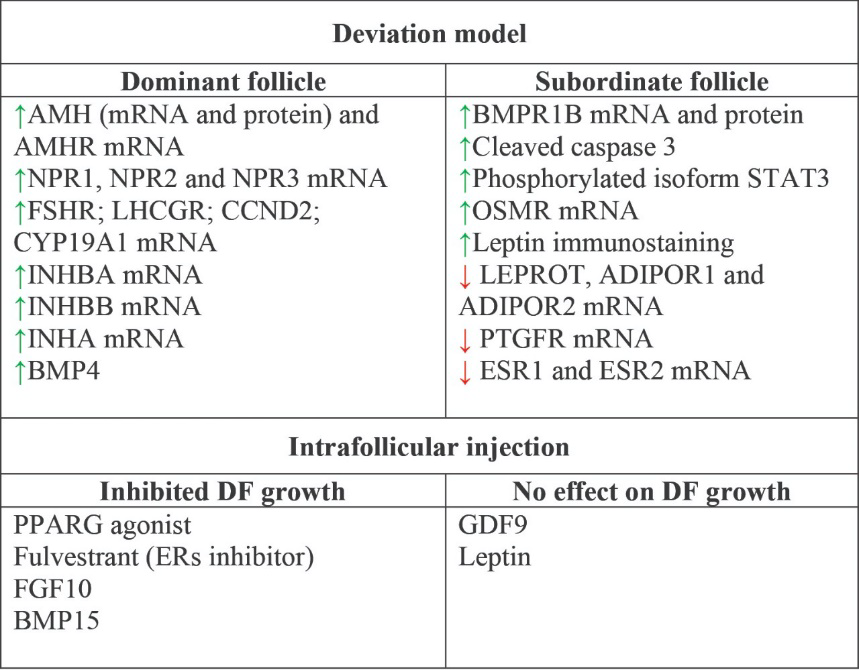
Summary of results from studies performed by our group investigating the local control of dominant follicle selection in cows. AMH: Anti-Mullerian hormone; AMHR: Anti-Mullerian hormone receptor; NPR: Natriuretic peptide receptor; FSHR: Follicle stimulating hormone receptor; LHCGR: Luteinizing hormone/choriogonadotropin receptor; CCND2: Cyclin D2; CYP19A1: Cytochrome P450 family 19 subfamily A member 1; INHBA: Inhibin subunit beta A; INHBB: Inhibin subunit beta B; INHA: Inhibin subunit alpha; BMP4: Bone morphogenetic protein 4; BMPR1B: Bone morphogenetic protein receptor type 1B; STAT3: Signal transducer and activator of transcription 3; OSMR: Oncostatin M receptor; LEPROT: Leptin receptor overlapping transcript; ADIPOR1: Adiponectin receptor 1; ADIPOR2: Adiponectin receptor 2; PTGFR: Prostaglandin F receptor; ESR1: Estrogen receptor 1; ESR2: Estrogen receptor 2; PPARG: Peroxisome proliferator activated receptor gamma; FGF10: Fibroblast growth factor 10; BMP15: Bone morphogenetic protein 15; GDF9: Growth differentiation factor 9; DF: Dominant Follicle; ERs: Estrogen receptors. Green arrow: Upregulation in granulosa cells (GC). Red arrow: Downregulation in GC.

**Figure 3:**
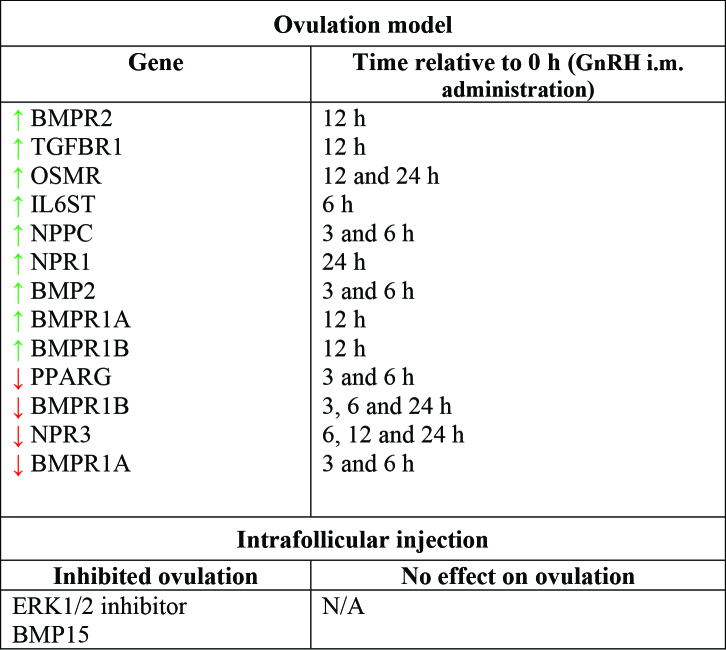
Summary of results from studies performed by our group investigating the local control of ovulation in cows. BMPR2: Bone morphogenetic protein receptor type 2; TGFBR1: Transforming growth factor beta receptor 1; OSMR: Oncostatin M receptor; IL6ST: Interleukin 6 cytokine family signal transducer; NPPC: Natriuretic peptide C; NPR1: Natriuretic peptide receptor 1; BMP2: Bone morphogenetic protein 2; BMPR1A: Bone morphogenetic protein receptor type 1A; BMPR1B: Bone morphogenetic protein receptor type 1B; PPARG: Peroxisome proliferator activated receptor gamma; NPR3: Natriuretic peptide receptor; ERK1/2: Extracellular signal-regulated protein kinase; BMP15: Bone morphogenetic protein 15. Green arrow: Upregulation in granulosa cells (GC). Red arrow: Downregulation in GC.

Besides the classical endocrine regulation of ovarian events, a complex local regulation of selection, follicular differentiation and ovulation processes has been revealed in recent decades. Characterization studies have revealed the regulation of the expression of genes encoding proteins and receptors during antral follicle development and ovulation. The deviation model allows to compare different features of the follicular environment in follicles before, during and after dominant follicle selection, while the ovulation model allows to investigate the acute changes induced between GnRH treatment and follicular rupture (Figure 1). A summary of the findings from our group regarding local factors during dominant follicle selection and ovulation is presented in Figure 2 and 3, respectively. Furthermore, through studies using the intrafollicular injection model, combined with studies involving immunization against proteins of interest, the functions of factors produced locally in the ovary have been revealed.

To date, scientific studies have shown that advances in understanding the local regulation will potentially lead to the discovery of new approaches to reverse reproductive disorders, predict the outcome of assisted reproduction techniques, increase the number of oocytes and/or embryos for superior genetic multiplication, obtain greater control of the moment of ovulation in the biotechniques of reproduction, promote contraception to control populations of domestic or wild animals. The present review discusses advances in the understanding of local factors involved in folliculogenesis and ovulation, focusing on data obtained by our group using *in vivo* models in bovine species. Further information regarding *in vivo* bovine models to study ovarian function can be assessed in a review by Rovani et al. (2018).

## Transforming growth factors: major players in the local control of antral follicle development

Factors produced in the ovary are considered regulators of the local follicular environment, determining the future of each follicle, and acting in different ovarian functions. The group of bone morphogenetic proteins (BMPs) is composed by about 20 ligands and seven serine/threonine receptor kinases (BMPRs) divided into type I and type II. These proteins, together with growth and differentiation factors (GDFs) and the anti-Mullerian hormone (AMH), belong to the superfamily of transforming growth factors beta (TGFβ; reviewed by Knight and Glister, 2006). TGFβ proteins initiate signaling by binding to a type II receptor, which in turn phosphorylates the kinase domain of a type I receptor. The phosphorylated type I receptor propagates the signal through the phosphorylation of proteins called SMADs, divided into three classes: regulated by the receptors (R-SMADs), the co-mediator (Co-SMAD) and the inhibitors (I-SMADs; Shi and Massagué, 2003). Activated SMAD complexes carry signaling to the nucleus and interact with nuclear cofactors to regulate transcription of target genes. GDF9, BMP15 (also known as GDF9b) and AMH are the main proteins of the TGFβ superfamily studied in ovarian function.

BMP15 and GDF9 may act separately in a monomeric way binding to type I and type II TGFβ receptors or synergistically, possibly as BMP15:GDF9 heterodimers (Heath et al., 2017). These proteins are expressed at all stages of follicular development in several animal species including bovine (Alam and Miyano, 2020) and have been shown to play central role in fertility (McNatty et al., 2007; Crawford and McNatty, 2012). Thus, interest in understanding the role of these factors in mammalian ovarian and reproductive physiology has increased in recent decades. In addition, studies demonstrate that the manipulation of TGFs can be applied in techniques to control the estrous cycle in different species such as sheep, cattle, horses, and deer (Galloway et al., 2000; Hanrahan et al., 2004; Juengel et al., 2009; Davis et al., 2018).

Oocyte-secreted BMP15 and GDF9 often act synergistically with surrounding cumulus, granulosa, and theca cells through paracrine mechanisms (Knight and Glister, 2006; Heath et al., 2017; Alam et al., 2018; Belli and Shimasaki, 2018). The functions attributed to BMP15 and GDF9 include regulation of follicular development, cumulus function, oocyte maturation, steroid production, expression of gonadotropin receptors, and determination of ovulatory rate (Paulini and Melo, 2011; Gasperin et al., 2014; Rossi et al., 2016). Furthermore, there is evidence of the involvement of these proteins in ovulation and luteinization processes (Juengel et al., 2009; Haas et al., 2022).

Functional studies investigating the role of oocyte-secreted proteins have been conducted in mice and ruminants. Infertility was observed in mice when the GDF9 gene was neutralized and follicular development did not progress from the primary follicle stage, in addition to decreasing granulosa cell proliferation (Dong et al., 1996). Mice neutralized for BMP15 also exhibit subfertility (Yan et al., 2001). In sheep, homozygous inactivating mutations in the genes encoding BMP15 (Galloway et al., 2000) and GDF9 (Hanrahan et al., 2004) have been identified as being associated with infertility. In this sense, in cows, immunization of GDF9 alone or in combination with BMP15 reduced follicular development and ovulation rate (Juengel et al., 2009). Therefore, GDF9 and BMP15 exert essential functions during follicular development in both mono- and poly-ovulatory species (Hanrahan et al., 2004; McNatty et al., 2004, 2007).

Besides follicular development, TGFβ superfamily factors act on mammalian ovulation. In sheep, point inactivating mutations in BMP15 and GDF9 (in heterozygosity), and in the BMPR1b (also known as ALK6), are associated with precocious follicular differentiation and increased ovulation rate (McNatty et al., 2005, 2004; Garcia-Guerra et al., 2018b). Juengel et al. (2009) performed active immunization against GDF9 and BMP15 proteins in cows and obtained superovulation in some animals, suggesting that these factors are involved in the selection of the dominant follicle and determination of the ovulation rate. In sheep, besides the increase in ovulation rate after a short period of immunization against BMP15 or GDF9, no significant effects were observed on oocyte fertilization, embryonic development and on pregnancy maintenance (Juengel et al., 2004). On the other hand, immunization against these proteins for prolonged periods caused a block in follicular development (McNatty et al., 2007). These results are different from those obtained in mares, in which immunization against GDF9 did not affect ovulation rate, but decreased preovulatory follicle diameter and estrus manifestation, while immunization against BMP15 negatively affected ovulatory rate and resulted in luteinization/ovulation of small follicles (Davis et al., 2018).

**Figure 4:**
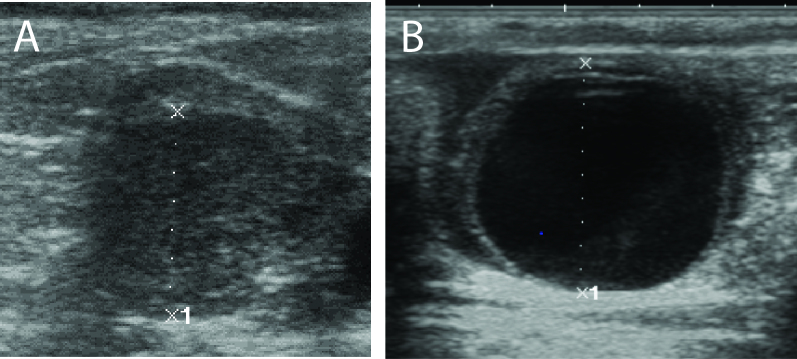
Corpus luteum with 23.2 mm 12 days after IFI with PBS (A). Luteal cyst with 31.6 mm 12 days after IFI with BMP15 (B). Adapted from Haas et al. (2022).

In cattle, using *in vivo* models, we have demonstrated that GDF9 concentration in follicular fluid does not differ between healthy and atretic follicles, and that GDF9 intrafollicular injection has no effect on dominant follicle growth (Haas et al., 2016). However, BMP15 intrafollicular injection inhibited dominant follicle growth and ovulation, but not luteinization, inducing the formation of luteinized cysts (Figure 4; Haas et al., 2022). Collectively, these results demonstrate a great potential for the use of BMP15 and/or GDF9 proteins regulation for increasing the number of selected dominant follicles and, as a consequence, increasing ovulation rate; or, paradoxically, to inhibit antral follicle development and ovulation, to promote contraception in domestic animals and humans.

Information regarding BMPs, GDFs and their receptors, has also been obtained using *in vitro* culture of granulosa and theca cells and/or from follicles obtained in slaughterhouses and classified according to the follicular diameter or concentration of estradiol in the follicular fluid. Experiments using granulosa cells culture have shown that the action of BMP15 and GDF9 appears to be synergistic and variable according to species (McNatty et al., 2005). The synthesis of BMP15 and GDF9 by oocytes starts during the transition from primordial to primary follicles (Knight and Glister, 2006; Sanfins et al., 2018). In this phase, BMP15 and GDF9 act synergistically to coordinate the proliferation and organization of granulosa cells (Knight & Glister, 2006; Paulini & Melo, 2011; Sanfins et al., 2018). In *in vitro* culture, BMP15 and GDF9 have also been shown to act synergistically to induce complexes of bovine granulosa cells to form antrum-like structures (Alam et al., 2018). However, in bovine granulosa cells cultured *in vitro* in a homologous system, increased expression of GDF9 and BMP15 caused cellular luteinization (Paulini and Melo, 2021), which resembles what we have observed when the intrafollicular concentration of BMP15 was increased in dominant and preovulatory follicles *in vivo*.

Besides the effects on follicular cells, GDF9 and BMP15 seem to be involved in oocyte developmental competence through a complex signaling process (Paulini & Melo, 2011; Alam et al., 2018; Belli & Shimasaki, 2018). Caixeta et al. (2009), searching for competence markers in bovine oocytes, observed high levels of BMP15 and GDF9 mRNA expression, but without regulation throughout antral follicular development. Donnison and Pfeffer (2004) observed higher expression of GDF9 mRNA in more competent bovine oocytes, whereas the expression of BMP15 did not differ between more or less competent oocytes. In human oocytes, studies report that GDF9 and BMP15 mRNA are significantly correlated with the oocyte maturation process (Li et al., 2014) and are strongly associated with cumulus cell proliferation, expansion, and development of cumulus-oocyte complexes of women and buffalo (Huang and Wells, 2010; Kathirvel et al., 2013). In mice, Park et al. (2020) demonstrated that ovarian expression of BMP15 and GDF9 decrease with age, indicating that these factors may serve as new biomarkers of ovarian aging. In this sense, mutations that affect BMP15 levels were identified as associated with early ovarian failure (Patiño et al., 2017). Therefore, BMP15 and GDF9 are fundamental for oocyte competence in different species, and influence cumulus function during follicular development and ovulation (Otsuka et al., 2011; Paulini and Melo, 2011).

Besides regulating cell proliferation and differentiation, several studies indicate that GDF9 and BMP15 act as regulators of steroidogenesis (Spicer et al., 2008). The addition of GDF9 in bovine theca cells culture decreases steroidogenesis stimulated by LH or IGF, by inhibiting the expression of steroidogenic enzymes (CYP11A1) and LH receptors (Spicer et al., 2008). A negative effect on estradiol synthesis was also observed after addition of GDF9 in granulosa cells culture treated with FSH and IGF (Spicer et al., 2006). When BMP15 was added to granulosa cells culture, it blocked the expression of FSH receptors (FSHR) and inhibited FSH-induced aromatase expression in the absence of androstenedione (Otsuka et al., 2001). Matsui et al. (2004) suggest the involvement of BMP15 in the process of follicular atresia, since BMP15 strongly inhibits FSH-stimulated pregnancy-associated protein (PAPP-A) expression in rat granulosa cells. In cases of inactivating mutations in sheep, reduced activity of growth factors induces early differentiation of developing follicles (Otsuka et al., 2001). The fact that granulosa cells from sheep heterozygous for the BMP15 inactivating mutation demonstrate greater LH responsiveness supports this hypothesis (McNatty et al., 2009).

## Regulation of BMP15 and GDF9 receptors and signaling

The signaling of BMP15 and GDF9 proteins is mediated by the BMPR2 receptor, differing only in the type I receptor, with BMPR1b (ALK6) being involved in BMP15 signaling, and TGFBR1 binding to GDF9. Kayani et al. (2009) demonstrated that BMPR1a, BMPR1b, BMPR2, ActR1A, ActR1B, ActR2A and ActR2B receptors are expressed on theca and granulosa cells. However, Glister et al. (2010) using follicles from slaughterhouses, classified according to diameter, showed little regulation of BMP receptor expression during follicular growth. Jayawardana et al. (2006) using pre and post selection follicles (mean 7.7 mm and 15 mm, respectively), demonstrated that the expression of BMPR2 and BMPR1a was higher in granulosa cells from post selection follicles. The same authors demonstrated that treatment with estradiol in granulosa cells obtained from follicles with approximately 4 mm of diameter increased the expression of BMPR2 and BMPR1a, and this expression was increased when combined with FSH. However, our *in vivo* studies have shown that BMPR2 and TGFRB1 receptor mRNA abundance is not differently regulated in granulosa cells of dominant and subordinate follicles (Gasperin et al., 2014). The same authors demonstrated that BMPR1b mRNA and protein are more abundant in granulosa cells from atretic subordinate follicles in cows, indicating a role during dominant follicle selection. In gilts, BMPR1b is downregulated after eCG+hCG treatment, indicating a role during luteinization (Hoyos-Marulanda et al., 2022). Therefore, it can be inferred that the BMPR1b receptor, in addition to regulating LH responsiveness, can also influence atresia. These results are in accordance with data obtained after active immunization in cows, which demonstrate that a partial decrease in BMP15 and GDF9 availability is associated with dominant follicle differentiation and increased ovulatory rate. Corroborating with this hypothesis, recently a high-fecundity allele named Trio has been described in cows, inducing upregulation of MAD family member 6 (SMAD6), an inhibitor of BMP/SMAD signaling pathway (Garcia-Guerra et al., 2018a). Cows carrying the Trio allele have multiple ovulations, with smaller preovulatory follicles and corpora lutea, compared to non-carriers, which resembles the features observed in ewes carrying mutations that increase ovulation rate.

Studies using *knockout* mice for the BMPR1a and BMPR1b have reported that these receptors have distinct functions during folliculogenesis, but act as suppressors of ovarian tumors (Edson et al., 2010). The same authors demonstrated that alterations in the normal pattern of expression of these receptors induce the formation of granulosa cell tumors. Tumor formations are also observed in the ovaries of ewes homozygous for the inactivating mutation of BMP15 (Juengel et al., 2000). Collectively, data available so far indicate that BMP15/GDF9 system is the main local regulator of follicle fate.

## Other members of the TGF superfamily

Besides GDF9 and BMP15, other BMPs have been studied in the mammalian ovary, but most studies are restricted to cell cultures and gene expression characterization. In bovine theca cell culture, BMPs 4, 6, and 7 inhibited androgen synthesis, decreasing the enzyme HSD17A (Glister et al., 2005), whereas an increase in IGF-stimulated E2 synthesis was observed in granulosa cells (Glister et al., 2004). In prepubertal ewe lambs, Juengel et al. (2006) observed low expression of BMPs 2, 4 and 7 in non-atretic follicles in all cell types evaluated, with high levels of BMP6 expression in the oocyte. Furthermore, in humans, it is assumed that BMP6 may be involved in the ovulatory process, since it is related to the suppression of protease and leukocytes inhibitors and increase in neutrophil attraction by granulosa cells cultured *in vitro* (Akiyama et al., 2014). In cattle, Kayani et al. (2009) demonstrated the inhibitory action of BMP6 on the expression of steroidogenic enzymes such as CYP11A1, HSD3B1 and CYP19A1 in granulosa cells cultured *in vitro*.

To elucidate the *in vivo* regulation in pre-ovulatory follicles, our group investigated the mRNA expression of BMPs and their receptors (unpublished data) in bovine granulosa cells after ovulation induction with GnRH. After 0, 3, 6, 12 or 24 hours of GnRH administration, the cows were ovariectomized and mRNA expression for BMPs 1, 2, 4, 6, and BMPR1a, BMPR1b, BMPR2 and TGFBR1 receptors in granulosa cells was analyzed. The results demonstrated that mRNA expression of BMPs 1 and 4 was not regulated by GnRH treatment. However, there was an increase in BMP2 expression at 3 and 6 h after GnRH, returning to baseline levels at 12 and 24 h. BMP6 expression was not detected in granulosa cell samples. The expression of BMPR1a and BMPR1b was highest at 0 h, decreasing at 3 and 6 h with a transient increase at 12 h. The expression of BMP2 and BMPR1a were positively associated with estradiol concentrations between LH surge and ovulation, while estradiol levels decreased as BMPR2 expression increased. Furthermore, mRNA expression of TGFBR1, BMPR1b and BMPR1a was negatively correlated with progesterone concentrations in follicular fluid. Therefore, the BMP system appears to play stimulatory and inhibitory paracrine actions in the local control of folliculogenesis and ovulation according to follicular stage.

The pattern of AMH expression in granulosa cells and follicular fluid around follicular deviation has also been evaluated by our group (Ilha et al., 2016). Interestingly, it was observed increased AMH and AMHR2 mRNA levels in dominant follicles at the expected time and after follicular deviation, compared to subordinate follicles. Furthermore, co-dominant follicles induced by FSH treatment had increased levels of AMH and AMHR mRNA (granulosa) and AMH protein in the follicular fluid. Thus, besides its well-established roles during preantral follicle development, our results suggest that AMH is involved in dominant follicle selection.

As described above, the functions of the TGFβ superfamily vary according to origin of the factor used, the species, and the type of cell studied. Furthermore, data obtained from *in vivo* and *in vitro* models are sometimes conflicting. Despite evidence indicating a potential use of immunization against BMP15 and GDF9 in reproductive control in different species, the mechanisms involved are still not fully understood. It is possible that the different effects observed are due to specific differences and variability in the humoral response by the different peptides or adjuvants used. Also, conflicting data in the literature suggest caution in extrapolating results among different species (Juengel et al., 2004, 2006; McNatty et al., 2005). Contradictions are observed even in studies carried out with the same species and may result from different cell culture conditions and degree of cell differentiation (Juengel et al., 2004).

## Conclusion

The identification of the participation of BMPs, GDF9 and related receptors in the local control of antral folliculogenesis is relatively recent. Many studies have been published using different species and models, often with contradictory results. The complete understanding of the function of these factors is far from being elucidated and for this purpose it is essential to use reliable models. Regulatory studies of this system should also include regulatory proteins. A greater understanding of this local regulatory system will enable an advance in the knowledge of physiology, ovarian disorders, as well as enabling the generation of technologies to increase the ovulatory rate or contraception in different species.
